# Supplementary material for: Population expansions shared among coexisting bacterial lineages are revealed by genetic evidence
Source: PeerJ. 2014 Dec 16;2:e696. doi: 10.7717/peerj.696 (PMC4273935; doi:10.7717/peerj.696)
Supplement: Supplemental Information 1 — Supplementary materials and methods [file peerj-02-696-s001.doc]

**SUPPLEMENTARY INFORMATION**

**Materials and Methods**

**Genetic diversity and neutrality tests**

Once populations were defined, genetic diversity analyses for populations within each lineage and gene were performed using DNAsp v4.1 (Rozas et al., 2003). Standard measures of nucleotide diversity (π) and the mutation parameter Waterson’s θ were estimated, together with neutrality tests (Tajima’s *D*, Fu and Li’s F* and D*, as well as F_S_). Briefly, both π and Waterson’s θ are measures of intraspecific genetic variation. The former is based on pairwise comparisons of nucleotide diversity and can be viewed as the average level of polymorphism, offering a description of the current condition of the population. By contrast, Waterson’s θ is based on the number of segregating sites present and incorporates the effective population size (N_e_) and thus it can be used to gain insight into historical demography (Hedrick, 2011). Tajima’s D (Tajima, 1989) evaluates discrepancies between π and Waterson’s θ. Under the Wright-Fisher neutral model (or mutation-drift model), π and Waterson’s θ are expected to be equal; deviations from neutrality can give information about population history, serving as a first approach to demographic events. Tests developed by Fu & Li (1993) analyze whether the frequency of singleton variants (restricted to the external or terminal branches (Hartl & Clark, 1997) are consistent with the mutation-drift expectation (Fu & Li 1993) and are considered a more direct method of analyzing background selection. In addition, Fu’s F_S_ (Fu, 1997) is sensitive to population growth and has been used as supporting evidence for demographic processes (Lessa, Cook & Patton, 2003). Significance for all neutrality tests was assessed using 10,000 coalescent simulations based on the observed number of segregating sites (Rozas et al., 2003).

**Figure S1**. ***16S rRNA*** **phylogenetic reconstructions of lineages studied**. Phylogenies were constructed using the maximum likelihood approach and the complete sequence of the 16S rRNA gene. **(a)** *Bacillus* lineages with *Geobacillus jurassicus* as outgroup, **(b)** *Exiguobacterium* lineages with *E. auranticum* as outgroup, and **(c)** *Pseudomona*s lineages with *Escherichia coli* as outgroup. Bold font denotes representative sequences of the lineages studied. The scale of the bar represents number of substitutions per site.

**Table S1**. PCR Primers used to amplify MLST loci in the three lineages studied.

**Table S2**. **Pairwise Homoplasy Index (*Φ*_W_) P values.** Values were obtained for all the housekeeping loci analyzed for each studied lineage using “PHI test recombination” function implemented in Splits Tree4 (Huson & Bryant, 2006). Bold fonts denote significant P values, which account for recombination. *NC* denotes non-computable estimations due to small number of informative sites. E1, E2 and E3 values were previously published in Rebollar et al. (2012).

**Table S3**. Pairwise *F_ST_* estimates. Values are shown for *Exiguobacterium* and *Bacillus* lineages. Bold font denotes significant *F_ST_* values (*P* < 0.05).

**Table S4. Summary statistics for genetic diversity and neutrality parameters for each population at each locus.** π is Nei’s pairwise nucleotide diversity value and θ is Watterson’s theta per site = 2Neμ; neutrality was tested using Tajima’s D, Fu & Li’s D* and F*, and Fu’s F_S_. Bold fonts denote significant values (*P* < 0.05) for neutrality tests (Rozas et al., 2003). N = number of sequences used in each analysis.

**Table S5.** Credibility intervals (95%) for estimates of number of population size changes. Values different from 0 (statistically supported expansions) are shown in bold.

**References**

Fu, Y.-X. (1997). Statistical Tests of Neutrality of Mutations Against Population Growth, Hitchhiking and Background Selection. *Genetics*, *147*, 915–925.

Fu, Y.-X., & Li, W.-H. (1993). Statistical Tests of Neutrality of Mutations. *Genetics*, *133*, 693–709.

Hartl, D. L., & Clark, A. G. (1997). *Principles of Population Geneticd* (3rd ed., p. 542). Sunderland, Massachussetts: Sinauer Associates, Inc.

Hedrick, P. W. (2011). *Genetics of Populations* (p. 553). Subdury, Massachusetts: Jones & Bartlett Publishers.

Huson, D. H., & Bryant, D. (2006). Application of Phylogenetic Networks in Evolutionary Studies. *Molecular Biology and Evolution*, *23*(2), 254–267.

Lessa, E. P., Cook, J. A., & Patton, J. L. (2003). Genetic footprints of demographic expansion in North America, but not Amazonia, during the Late Quaternary. *Proceedings of the National Academy of Sciences of the United States of America*, *100*(18), 10331–4. doi:10.1073/pnas.1730921100

Rebollar, E. a, Avitia, M., Eguiarte, L. E., González-González, A., Mora, L., Bonilla-Rosso, G., & Souza, V. (2012). Water-sediment niche differentiation in ancient marine lineages of Exiguobacterium endemic to the Cuatro Cienegas Basin. *Environmental Microbiology*, *14*(9), 2323–2333. doi:10.1111/j.1462-2920.2012.02784.x

Rozas, J., Sanchez-DelBarrio, J. C., Messeguer, X., & Rozas, R. (2003). DnaSP, DNA polymorphism analyses by the coalescent and other methods. *Bioinformatics*, *19*(18), 2496–2497. doi:10.1093/bioinformatics/btg359

Tajima, F. (1989). Statistical method for testing the neutral mutation hypothesis by DNA polymorphism. *Genetics*, *123*, 585–595.
