# Supplementary figures and images for: Population expansions shared among coexisting bacterial lineages are revealed by genetic evidence
Source: PeerJ. 2014 Dec 16;2:e696. doi: 10.7717/peerj.696 (PMC4273935; doi:10.7717/peerj.696)

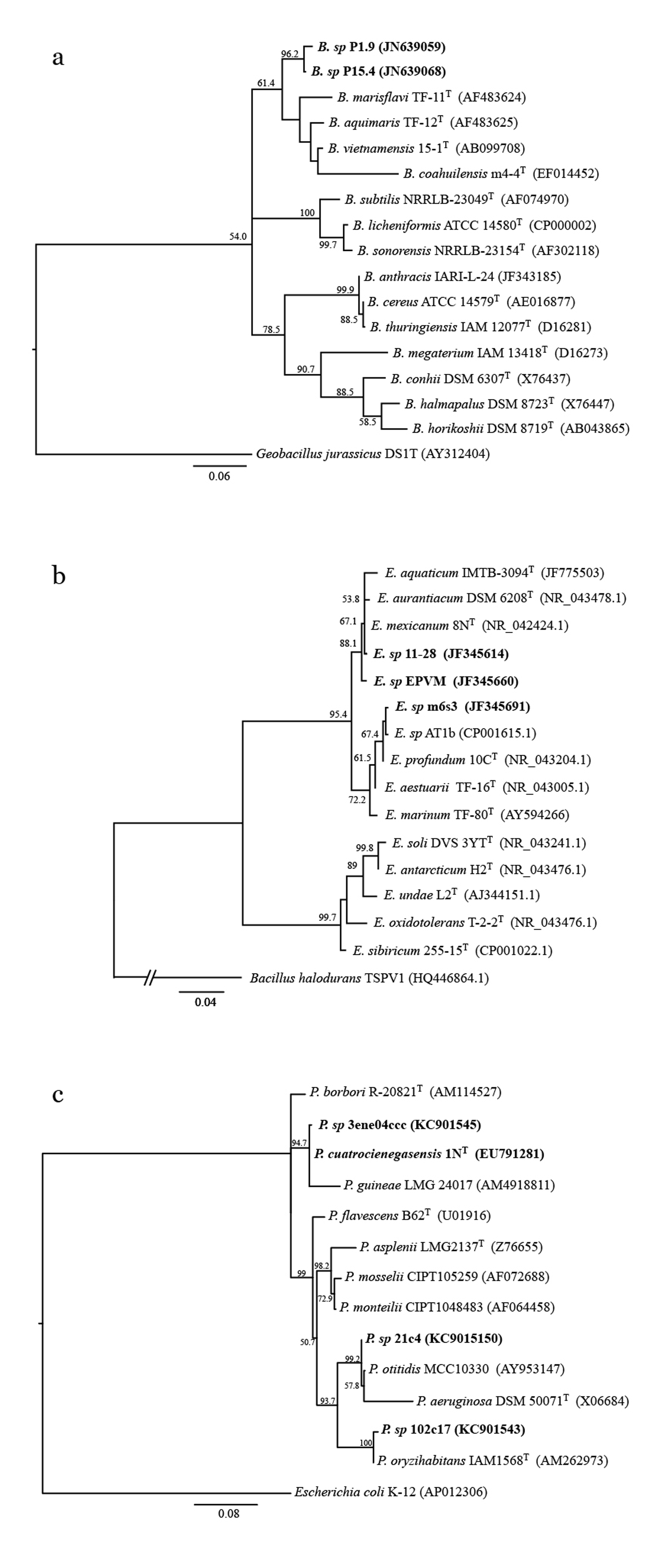

Supplement: Figure S1 — Phylogenies were constructed using the maximum likelihood approach and the complete sequence of the 16S rRNA gene. (a) Bacillus lineages with Geobacillus jurassicus as outgroup, (b) Exiguobacterium lineages with E. auranticum as outgroup, and (c) Pseudomonas lineages with Escherichia coli as outgroup. Bold font denotes representative sequences of the lineages studied. The scale of the bar represents number of substitutions per site. [file peerj-02-696-s002.png]
