# Supplementary material for: Population expansions shared among coexisting bacterial lineages are revealed by genetic evidence
Source: PeerJ. 2014 Dec 16;2:e696. doi: 10.7717/peerj.696 (PMC4273935; doi:10.7717/peerj.696)
Supplement: Table S1 — Primer sequences obtained from previous publications and this study are indicated. [file peerj-02-696-s003.doc]

**Table S1. PCR Primers used to amplify MLST loci in the three lineages studied.**

| Genus | Locus | Primers | T_m_(°C) | Sequence (5´-3´) | PCR product size (bp) | Reference |
| --- | --- | --- | --- | --- | --- | --- |
| *Exiguobacterium* | *citC* | citC-F | 49 | GGDGAYGGMACWGGWCCWGAYATTTGG | 1,165 | Rodrigues, 2006 |
|  |  | citC-R |  | AATTCWGAACATTTMACTTCTGT |  | Rodrigues, 2006 |
|  | *hsp70* | hsp70-F | 47 | GGTATTGAYTTAGGAACAACAAACT | 1455 | Rodrigues, 2006 |
|  |  | hsp70-R |  | CTTCTGCWTCTTTKACCAT |  | Rodrigues, 2006 |
|  | *recA* | recA-F | 47 | GARAARCAATTYGGBAAAGGTTC | 869 | Rodrigues, 2006 |
|  |  | recA-R |  | TGTTTYGMATTTTCACGKCCTTG |  | Rodrigues, 2006 |
|  | *rpoB* | rpoB-F | 53 | CGAACATGCAACGTCAGGC | 1078 | Rodrigues, 2006 |
|  |  | rpoB-R |  | ACATCYTCYTCACGNGCACC |  | Rodrigues, 2006 |
| *Pseudomonas* | *acnB* | acn-Fp | 60 | ACATCCCGCTGCACGCYCTGGCC | 700 | Sarkar and Guttman 2004 |
|  |  | acn-Rp |  | GTGGTGTCCTGGGAACCGACGGTG |  | Sarkar and Guttman 2004 |
|  | *gyrB* | gyrB_F | 60 | ACCAYGSNGGNGGNAARTTYRA | 1000 | Modified from Yamamoto *et al*. 2000 |
|  |  | gyrB_R |  | AGTGCNGGRTCYTTYTCYTGRCA |  | Modified from Yamamoto *et al*. 2000 |
|  | *recA* | recA-F | 60 | CAGATCGAAMRNCARTTCGG | 900 | This study^a^ |
|  |  | recA-R |  | YTTRCCCTGDCCGATCTT |  | This study^a^ |
|  | *rpoD* | 70F | 63 | TGTAYATGMGNGARATGGGNACNGT | 850 | Modified from Yamamoto *et al*. 2000 |
|  |  | 70R |  | GTTNGCYTCNACCATYTCYTTYTT |  | Modified from Yamamoto *et al*. 2000 |
| *Bacillus* | *citC* | citC-F | 52-54.5 | GGDGAYGGMACWGGWCCWGAYATTTGG | 1,165 | Rodrigues, 2006 |
|  |  | citC-R |  | AATTCWGAACATTTMACTTCTGT |  | Rodrigues, 2006 |
|  | *gltX* | gltx-F | 52 | CGYGGBGADGAYCAYATYT | 488 | This study^a^ |
|  |  | gltX-R |  | CRATTTCMGCDCCRWARCT |  | This study^a^ |
|  | *hsp70* | hsp70-F | 52.5-54.5 | GGTATTGAYTTAGGAACAACAAACT | 1455 | Rodrigues, 2006 |
|  |  | hsp70-R |  | CTTCTGCWTCTTTKACCAT |  | Rodrigues, 2006 |
|  | *recA* | recA-F | 51 | GATCGTCARGCAGSCYTWGAT | 574 | This study^a^ |
|  |  | recA-R |  | TTWCCRACCATAACSCCRAC |  | This study^a^ |
|  | *spo0A* | spo0A-F1 | 49-52 | GATGAYAATCGCGAGYTKGTH | 505 | This study^a^ |
|  |  | spo0A-F2 |  | TWDTDGATGAYAATCGNGARY | 511 | This study^a^ |
|  |  | spo0A-R |  | KARATAACCYTTATTRTGGGC |  | This study^a^ |

^a^ Primers specifically designed for this study from sequences obtained from GenBank.
