# Supplementary material for: Population expansions shared among coexisting bacterial lineages are revealed by genetic evidence
Source: PeerJ. 2014 Dec 16;2:e696. doi: 10.7717/peerj.696 (PMC4273935; doi:10.7717/peerj.696)
Supplement: Table S2 — Values were obtained for all the housekeeping loci analyzed for each studied lineage using “PHI test recombination” function implemented in Splits Tree4 (Huson & Bryant, 2006). Bold fonts denote significant P values, which account for recombination. NC denotes non-computable estimations due to small number of informative sites. E1, E2 and E3 values were previously published in Rebollar et al. (2012). [file peerj-02-696-s004.doc]

**Table S2**. **Pairwise Homoplasy Index (*Φ*_W_) P values** obtained for all the housekeeping loci analyzed for each studied lineage using “PHI test recombination” function implemented in Splits Tree4 (Huson & Bryant, 2006). Bold fonts denote significant P values, which account for recombination. *NC* denotes non-computable estimations due to small number of informative sites. E1, E2 and E3 values were previously published in Rebollar et al. (2012).

|  | | **Genetic marker** | ***Φ*_W_ P value** |
| --- | --- | --- | --- |
| ***Bacillus*** | **B1** | *citC* | 0.58 |
|  |  | *gltX* | 0.375 |
|  |  | *hsp70* | 0.199 |
|  |  | *recA* | 0.254 |
|  |  | *spo0A* | 0.261 |
|  | **B2** | *citC* | 0.057 |
|  |  | *gltX* | NC |
|  |  | *hsp70* | 1 |
|  |  | *recA* | 1 |
|  |  | *spo0A* | 1 |
| ***Exiguobacterium*** | **E1** | *citC* | 0.317 |
|  |  | *hsp70* | 0.927 |
|  |  | *recA* | 1 |
|  |  | *rpoB* | 1 |
|  | **E2** | *citC* | **0.001** |
|  |  | *hsp70* | 0.184 |
|  |  | *recA* | **0.028** |
|  |  | *rpoB* | 0.828 |
|  | **E3** | *citC* | 0.346 |
|  |  | *hsp70* | 0.29 |
|  |  | *recA* | 0.404 |
|  |  | *rpoB* | 0.108 |
| ***Pseudomonas*** | **P1** | *acnB* | 0.9157 |
|  |  | *gyrB* | **0.039** |
|  |  | *recA* | 0.769 |
|  |  | *rpoD* | 1 |
|  | **P2** | *acnB* | 0.123 |
|  |  | *gyrB* | 0.891 |
|  |  | *recA* | 0.064 |
|  |  | *rpoD* | 0.189 |
|  | **P3** | *acnB* | 1 |
|  |  | *gyrB* | NC |
|  |  | *recA* | NC |
|  |  | *rpoD* | NC |
