# Supplementary material for: Population expansions shared among coexisting bacterial lineages are revealed by genetic evidence
Source: PeerJ. 2014 Dec 16;2:e696. doi: 10.7717/peerj.696 (PMC4273935; doi:10.7717/peerj.696)
Supplement: Table S3 — Values are shown for Exiguobacterium and Bacillus lineages. Bold font denotes significant FST values (P < 0.05). [file peerj-02-696-s005.doc]

**Table S3**. Pairwise *F_ST_* estimates for *Exiguobacterium* and *Bacillus* lineages. Bold font denotes significant *F_ST_* values (*P* < 0.05).

| ***Bacillus* B1** |  |  |  |  |
| --- | --- | --- | --- | --- |
|  | Churince | Los Hundidos | Mesquites | Pozas Azules |
| Churince | 0 |  |  |  |
| Los Hundidos | **0.13122** | 0 |  |  |
| Mesquites | **0.05384** | **0.05815** | 0 |  |
| Pozas Azules | **0.06417** | 0.01184 | **0.02442** | 0 |

| ***Bacillus* B2** |  |  |  |  |
| --- | --- | --- | --- | --- |
|  | Churince | Mesquites | Pozas Azules |  |
| Churince | 0 |  |  |  |
| Mesquites | **0.07361** | 0 |  |  |
| Pozas Azules | -0.08534 | -0.06249 | 0 |  |

| ***Exiguobacterium* E1** | |  |  |  |
| --- | --- | --- | --- | --- |
|  | Churince | Los Hundidos | Mesquites | Pozas Azules |
| Churince | 0 |  |  |  |
| Los Hundidos | -0.00447 | 0 |  |  |
| Mesquites | **0.15847** | **0.19081** | 0 | **0.18473** |
| Pozas Azules | **0.07145** | 0.04933 |  | 0 |

| ***Exiguobacterium* E2** |  |  |  |  |
| --- | --- | --- | --- | --- |
|  | Churince | Los Hundidos | Mesquites |  |
| Churince | 0 |  |  |  |
| Los Hundidos | 0.05388 | 0 |  |  |
| Mesquites | **0.19335** | **0.3549** | 0 |  |

| ***Exiguobacterium* E3** |  |  |  |  |
| --- | --- | --- | --- | --- |
|  | Churince | Mesquites |  |  |
| Churince | 0 |  |  |  |
| Mesquites | -0.01191 | 0 |  |  |
