# Supplementary material for: Population expansions shared among coexisting bacterial lineages are revealed by genetic evidence
Source: PeerJ. 2014 Dec 16;2:e696. doi: 10.7717/peerj.696 (PMC4273935; doi:10.7717/peerj.696)
Supplement: Table S4 — π is Nei’s pairwise nucleotide diversity value and θ is Watterson’s theta per site = 2Neµ; neutrality was tested using Tajima’s D, Fu & Li’s D* and F*, and Fu’s FS. Bold fonts denote significant values (P < 0.05) for neutrality tests (Rozas et al., 2003). N = number of sequences used in each analysis. [file peerj-02-696-s006.doc]

**Table S4. Summary statistics for genetic diversity and neutrality parameters for each population at each locus.** π is Nei’s pairwise nucleotide diversity value and θ is Watterson’s theta per site = 2Neμ; neutrality was tested using Tajima’s D, Fu & Li’s D* and F*, and Fu’s F_S_. Bold fonts denote significant values (*P* < 0.05) for neutrality tests (Rozas et al., 2003). N = number of sequences used in each analysis.

|  |  | **Genetic marker (N)** | **π** | **θ** | **D** | **D*** | **F*** | **F_S_** |
| --- | --- | --- | --- | --- | --- | --- | --- | --- |
| ***Bacillus*** | **B1_C** | *citC*(13) | 0.00634 | 0.00659 | -0.14865 | 0.20065 | 0.12398 | -0.001 |
|  |  | *gltx*(13) | 0.02418 | 0.01665 | 1.93412 | 1.55213 | 1.88975 | 6.931 |
|  |  | *hsp70*(13) | 0.00676 | 0.00917 | -1.09708 | -1.08285 | -1.24064 | -1.684 |
|  |  | *recA*(13) | 0.00579 | 0.00546 | 0.24002 | 0.95617 | 0.87547 | -2.14 |
|  |  | *spo0A*(13) | 0.00677 | 0.00992 | -1.29439 | -1.33265 | -1.50892 | -0.415 |
|  | **B1_HPa** | *citC*(63) | 0.00636 | 0.00911 | -0.93631 | **-2.8527** | **-2.57788** | **-9.203** |
|  |  | *gltx*(63) | 0.01236 | 0.01481 | -0.52713 | -0.37074 | -0.50997 | -1.966 |
|  |  | *hsp70*(63) | 0.00388 | 0.00765 | **-1.5085** | -0.78297 | -1.23732 | **-5.974** |
|  |  | *recA*(63) | 0.00482 | 0.00889 | -1.42751 | **-3.78042** | **-3.49337** | -3.499 |
|  |  | *spo0A*(63) | 0.00792 | 0.01796 | **-1.81687** | **-4.26053** | **-4.00576** | -4.839 |
|  | **B1_M** | *citC*(29) | 0.00584 | 0.00677 | -0.45529 | -0.68539 | -0.71918 | -3.595 |
|  |  | *gltx*(29) | 0.0124 | 0.01842 | -1.18276 | -1.76197 | -1.85484 | -1.998 |
|  |  | *hsp70*(29) | 0.00459 | 0.00918 | **-1.7444** | -1.6035 | -1.93523 | **-7.155** |
|  |  | *recA*(29) | 0.00212 | 0.00432 | **-1.59693** | -0.36113 | -0.86063 | **-4.852** |
|  |  | *spo0A*(29) | 0.00976 | 0.0111 | -0.41454 | -0.88609 | -0.8659 | **-13.094** |
|  | **B2_C** | *citC*(9) | 0.00329 | 0.00376 | -0.52629 | 0.04471 | -0.10194 | -1.338 |
|  |  | *gltx*(9) | 0.00129 | 0.00095 | 0.98627 | 0.8404 | 0.96219 | 0.849 |
|  |  | *hsp70*(9) | 0.00285 | 0.00279 | 0.07803 | 0.50315 | 0.44642 | -0.208 |
|  |  | *recA*(9) | 0.00149 | 0.00226 | -0.88249 | -0.7022 | -0.88249 | -2.106 |
|  |  | *spo0A*(9) | 0.001 | 0.00094 | 0.15647 | 0.8404 | 0.74837 | 0.477 |
|  | **B2_MPa** | *citC*(37) | 0.0029 | 0.00392 | -0.75486 | -0.02029 | -0.29221 | **-5.51971** |
|  |  | *gltx*(37) | 0.00135 | 0.00124 | 0.17481 | -0.80429 | -0.60451 | 0.285 |
|  |  | *hsp70*(37) | 0.001 | 0.00182 | -1.09093 | -1.06705 | -1.25012 | -2.126 |
|  |  | *recA*(37) | 0.00126 | 0.00208 | -1.51297 | -1.68268 | -1.82046 | -0.38 |
|  |  | *spo0A*(37) | 0.00216 | 0.00307 | -0.77205 | -0.7022 | -0.84352 | **-4.305** |
| ***Exiguobacterium*** | **E1_CHPa** | *citC*(69) | 0.00499 | 0.01563 | **-2.25866** | **-6.22055** | **-5.61834** | 1.465 |
|  |  | *hsp70*(69) | 0.00544 | 0.02513 | **-2.66051** | **-5.27413** | **-5.08687** | -1.285 |
|  |  | *recA*(68) | 0.01998 | 0.02007 | -0.01436 | -1.7318 | -1.2783 | 1.678 |
|  |  | *rpoB*(68) | 0.00128 | 0.00533 | -2.22123 | **-4.3899** | **-4.3083** | -5.028 |
|  | **E1_M** | *citC*(63) | 0.00599 | 0.00375 | 1.69688 | -0.80621 | 0.0581 | 3.985 |
|  |  | *hsp70*(64) | 0.00127 | 0.00279 | -1.45722 | **-2.82187** | **-2.79375** | -2.175 |
|  |  | *recA*(62) | 0.01022 | 0.0382 | **-2.54278** | **-6.32914** | **-5.81342** | 2.901 |
|  |  | *rpoB*(64) | 0.00113 | 0.00326 | **-1.74904** | **-4.20979** | **-4.00043** | -2.069 |
|  | **E2_CH** | *citC*(16) | 0.03332 | 0.02845 | 0.73142 | 0.5417 | 0.68842 | 7.808 |
|  |  | *hsp70*(16) | 0.04698 | 0.03728 | 1.1183 | 1.20473 | 1.36438 | 7.98 |
|  |  | *recA*(16) | 0.0249 | 0.02303 | 0.34381 | 0.42753 | 0.4665 | 3.896 |
|  |  | *rpoB*(16) | 0.02074 | 0.02193 | -0.22758 | -0.50261 | -0.49062 | 0.955 |
|  | **E2_M** | *citC*(12) | 0.02912 | 0.03077 | -0.24782 | -0.23508 | -0.2719 | 8.152 |
|  |  | *hsp70*(12) | 0.04065 | 0.04328 | -0.28249 | -0.1994 | -0.25244 | 10.151 |
|  |  | *recA*(12) | 0.0229 | 0.02467 | -0.3278 | -0.27522 | -0.32949 | 1.598 |
|  |  | *rpoB*(12) | 0.01538 | 0.01778 | -0.60888 | -0.19975 | -0.34979 | 4.372 |
|  | **E3_CM** | *citC* (19) | 0.02814 | 0.0169 | 2.68291 | 2.683 | 2.08208 | 10.933 |
|  |  | *hsp70* (19) | 0.0169 | 0.00932 | 0.53515 | -0.24331 | -0.01875 | 1.08 |
|  |  | *recA* (19) | 0.02018 | 0.01753 | 0.60746 | 1.0072 | 1.03414 | 6.199 |
|  |  | *rpoB* (19) | 0.00471 | 0.047 | 0.0054 | -0.45778 | -0.37705 | -1.132 |
| ***Pseudomonas*** | **P1_C** | *acnB* (16) | 0.01041 | 0.01874 | **-1.85733** | **-2.05057** | **-2.3048** | **-4.793** |
|  |  | *gyrB* (15) | 0.02787 | 0.02237 | 1.065 | 0.74481 | 0.96143 | 2.495 |
|  |  | *recA* (5) | 0.03062 | 0.03349 | -0.647 | -0.96758 | -0.97904 | 0.717 |
|  |  | *rpoD* (16) | 0.00426 | 0.00556 | -0.88876 | -1.56786 | -1.58834 | 1.2 |
|  | **P2_C** | *acnB* (26) | 0.00909 | 0.01869 | **-1.94317** | **-2.64783** | **-2.84909** | **-13.503** |
|  |  | *gyrB* (15) | 0.00816 | 0.00867 | -0.2464 | -0.16294 | -0.21393 | -2.4 |
|  |  | *recA* (17) | 0.01131 | 0.01487 | -0.96926 | -1.81228 | -1.8181 | **-5.679** |
|  |  | *rpoD* (28) | 0.00341 | 0.00451 | -0.76648 | -0.34344 | -0.55018 | -2.886 |
|  | **P3_C** | *acnB* (7) | 0.02005 | 0.01953 | 0.15214 | 0.61134 | 0.56146 | -1.169 |
|  |  | *gyrB* (15) | 0.00032 | 0.00073 | -1.49051 | -1.87275 | -2.0195 | 0.235 |
|  |  | *recA* (6) | 0.00248 | 0.00326 | -1.36732 | -1.39992 | -1.48974 | **-2.112** |
|  |  | *rpoD* (7) | 0.00093 | 0.00132 | **-1.23716** | -1.29591 | -1.37408 | -0.922 |
