# Supplementary material for: Population expansions shared among coexisting bacterial lineages are revealed by genetic evidence
Source: PeerJ. 2014 Dec 16;2:e696. doi: 10.7717/peerj.696 (PMC4273935; doi:10.7717/peerj.696)
Supplement: Table S5 — Values different from 0 (statistically supported expansions) are shown in bold. [file peerj-02-696-s007.docx]

| Lineage_population | Mean | 95% HPD lower | 95% HPD higher |
| --- | --- | --- | --- |
| E1_CHPa | 0.428 | 0 | 2 |
| **E1_M** | **2.3** | **1** | **4** |
| E2_M | 0 | na | na |
| **E2_CH** | **1.94** | **1** | **3** |
| E3_CM | 1.16 | 0 | 3 |
| B1_HPa | 0.679 | 0 | 2 |
| B1_M | 2.60E-2 | 0 | 0 |
| B1_C | 0 | na | na |
| **B2_MPa** | **1.47** | **1** | **3** |
| B2_C | 0.786 | 0 | 3 |
| P1_C | 0.817 | 0 | 2 |
| **P2_C** | **1.355** | **1** | **3** |
| P3_C | 1.58 | 0 | 3 |

**Table S5**. Credibility intervals (95%) for estimates of number of population size changes. Values different from 0 (statistically supported expansions) are shown in bold.
